# Twenty-eight day repeated exposure of human 3D bronchial epithelial model to heated tobacco aerosols indicates decreased toxicological responses compared to cigarette smoke

**DOI:** 10.3389/ftox.2023.1076752

**Published:** 2023-02-16

**Authors:** Fiona Chapman, Sarah Jean Pour, Roman Wieczorek, Edgar Trelles Sticken, Jessica Budde, Karin Röwer, Sandra Otte, Elizabeth Mason, Lukasz Czekala, Thomas Nahde, Grant O’Connell, Liam Simms, Matthew Stevenson

**Affiliations:** ^1^ Imperial Brands PLC, Bristol, United Kingdom; ^2^ Reemtsma Cigarettenfabriken GmbH, Hamburg, Germany

**Keywords:** *in vitro*, MucilAir™, heated tobacco, cigarette, tobacco harm reduction, repeat exposure

## Abstract

Tobacco harm reduction (THR) involves providing adult smokers with potentially reduced harm modes of nicotine delivery as alternatives to smoking combustible cigarettes. Heated tobacco products (HTPs) form a category with THR potential due to their ability to deliver nicotine and flavours through heating, not burning, tobacco. By eliminating burning, heated tobacco does not produce smoke but an aerosol which contains fewer and lower levels of harmful chemicals compared to cigarette smoke. In this study we assessed the *in vitro* toxicological profiles of two prototype HTPs’ aerosols compared to the 1R6F reference cigarette using the 3D human (bronchial) MucilAir™ model. To increase consumer relevance, whole aerosol/smoke exposures were delivered repeatedly across a 28 day period (16, 32, or 48 puffs per exposure). Cytotoxicity (LDH secretion), histology (Alcian Blue/H&E; Muc5AC; FoxJ1 staining), cilia active area and beat frequency and inflammatory marker (IL-6; IL-8; MMP-1; MMP-3; MMP-9; TNFα) levels were assessed. Diluted 1R6F smoke consistently induced greater and earlier effects compared to the prototype HTP aerosols across the endpoints, and in a puff dependent manner. Although some significant changes across the endpoints were induced by exposure to the HTPs, these were substantially less pronounced and less frequently observed, with apparent adaptive responses occurring over the experimental period. Furthermore, these differences between the two product categories were observed at a greater dilution (and generally lower nicotine delivery range) for 1R6F (1R6F smoke diluted 1/14, HTP aerosols diluted 1/2, with air). Overall, the findings demonstrate the THR potential of the prototype HTPs through demonstrated substantial reductions in toxicological outcomes in *in vitro* 3D human lung models.

## Introduction

Combustible cigarette smoking is cause of serious diseases, including heart disease, lung cancer and emphysema (United States Surgeon General, 2010; International Agency for Research on Cancer, 2012; US Department of Health and Human Services, 2014). This is largely attributed to the repeated exposure of cells to a vast number of toxicants within the smoke generated upon the combustion of tobacco, which in turn can cause the initiation of cellular and molecular processes leading to disease (US Department of Health and Human Services, 2014). Tobacco harm reduction (THR) is the concept of providing adult smokers who would otherwise continue to smoke with potentially less harmful forms of nicotine delivery (Stratton et al., 2001; O’Leary and Polosa, 2020). This is achieved through offering adult smokers nicotine delivery innovations, or next-generation products (NGPs), which deliver satisfactory levels of nicotine but with substantially reduced levels of, and fewer, toxicants compared to combustible cigarette smoke (O’Leary and Polosa, 2020). Further to this, nicotine delivery products are proposed to sit on a product risk scale relative to one another, with medically licenced nicotine replacement therapies, such as gum and patches, at the lower risk end, combustible cigarette smoking at the highest risk by a large margin, and NGPs in between (McNeill and Munafò, 2013; Abrams et al., 2018; Zeller, 2019; Murkett et al., 2020). NGPs which deliver nicotine *via* the inhalation route and do not involve tobacco combustion include e-cigarettes and heated tobacco products (HTPs). By eliminating burning, these products do not produce smoke but an inhalable aerosol which contains fewer and lower levels of potentially harmful chemicals (Forster et al., 2018; Bentley et al., 2020; Rudd et al., 2020; Chapman et al., 2023).

Increasing evidence in the scientific literature suggests that the reduction in levels and number of toxicants within the aerosols of NGPs, including e-cigarettes and heated tobacco, correlates with reductions in *vitro* toxicological responses compared to combustible cigarette smoke (Schaller et al., 2016; Jaunky et al., 2018; Hattori et al., 2020; Rudd et al., 2020; Simms et al., 2020; Simms et al., 2021; Chapman et al., 2023). Furthermore, the substantially reduced toxicological responses of NGPs compared to combustible cigarette has been demonstrated in a number of *in vitro* studies, in both 2D and 3D cell models (Iskandar et al., 2018; Czekala et al., 2019; Czekala et al., 2020; Haswell et al., 2021; Simms et al., 2021; Simms et al., 2022). Three dimensional (human) lung cell models are a useful tool in the assessment of the effects of inhalable test articles such as combustible cigarette smoke or NGP aerosols (Bedford et al., 2022). These models offer a human-relevant cellular system, grown and/or stimulated under air-liquid interface (ALI) conditions, and can include a variety of relevant cell types (e.g., basal, goblet and ciliated cells); this is more closely representative of an *in vivo* scenario than using single cell-type 2D cultures, for example (Huang et al., 2013; Cervena et al., 2019). This, coupled with the application of whole aerosol/smoke exposures at the ALI can model exposure of cells to as representative a chemical mixture to that which the consumer would be exposed to as possible (Chapman et al., 2023). *In vitro* 3D models exposed to whole NGP aerosol or combustible cigarette smoke have been used to assess a number of endpoints associated with the development and progression of respiratory diseases, including functional and morphological changes like cilia activity and ratios of cell types (e.g., goblet cells, ciliated cells), inflammatory readouts and genomic level changes (Czekala et al., 2019; Rayner et al., 2019; Ghosh et al., 2020; Haswell et al., 2021; Bedford et al., 2022).

Most of the previous *in vitro* studies utilising human 3D lung tissue models in the assessment of the effects of NGPs compared to combustible cigarettes have applied acute (single) exposures (Iskandar et al., 2018; Czekala et al., 2019; Giralt et al., 2021). However, it is recognised that development of disease phenotypes occurs following a period of time, and additionally is likely the effect of more than a single exposure or cellular/molecular event (Yoshida and Tuder, 2007; Haswell et al., 2021; Luettich et al., 2021). Furthermore, upregulation of responses to exogenous agents may have protective effects in the event of any subsequent exposures, or may result in increased susceptibility and instability of cellular processes (Chapman et al., 2015). Czekala et al. (2021) recently compared the effects of repeat exposures of diluted fresh whole 3R4F reference cigarette smoke to whole fresh undiluted e-cigarette aerosol and found that whilst 3R4F induced strong declines in cellular functionality and integrity over a 28 days repeated exposure period compared to Sham, the e-cigarette did not. At lower exposures repeated over a 6 week period, diluted (1:80, then later 1:100) 1R6F reference cigarette smoke induced increases in the population of mucin-producing cells, or goblet cell hyperplasia, a pathology associated with development of COPD (Haswell et al., 2021). Crucially, application of repeat exposures, over a long-term period, is more representative of a consumer relevant scenario, i.e., repeated, regular product use. The 3D MucilAir model is considered a useful tool in such repeat exposure studies as it can be maintained in the incubator in its fully differentiated state for up to 1 year (Cervena et al., 2019; Epithelix Sàrl, 2022). However, studies on repeat exposure to NGPs compared to combustible cigarettes *in vitro* are limited, particularly with regards to HTPs.

Mucociliary clearance, which involves both cilia activity and the airway surface liquid (including mucus and the periciliary layer) play a key role in maintenance of airway functionality, and dysfunction is linked to the development of pathologies such as COPD (Luettich et al., 2021). This adverse event pathway, which may occur upon exposure to combustible cigarette smoke, has recently been mapped out by Luettich et al. (2021). As part of this pathway, FoxJ1 has a role in ciliogenesis, and its decreased expression, which has been linked with combustible cigarette smoke exposure, is associated with loss/absence of cilia (Luettich et al., 2021). Goblet cell hyperplasia is another process associated with respiratory disease pathologies, and has recently been modelled *in vitro* during sub-cytotoxic repeated exposures to diluted combustible cigarette smoke (but was not induced by HTP aerosol exposure) (Haswell et al., 2021); Muc5AC is often used as a marker for mucin gene expression (Behrsing et al., 2016; Bedford et al., 2022). Additionally, changes in inflammatory markers are used as a proxy of inflammatory responses *in vitro*, and a number of mediators, including the cytokines, TNFα and interleukins (ILs), and matrix metalloproteinases (MMPs), are associated with exposure to inhaled toxicants, including combustible cigarette smoke (Behrsing et al., 2016; Czekala et al., 2021; Bedford et al., 2022; Langel et al., 2022). It is for the above reasons that these cellular processes (cilia activity (beat frequency and active area), FoxJ1 expression, changes in goblet cells, inflammatory readouts) are often used as *in vitro* indicators of potential responses to inhaled test articles (Behrsing et al., 2016; Iskandar et al., 2017; Czekala et al., 2021; Bedford et al., 2022). Therefore in the current study, we looked to assess these endpoints.

The current study aimed to assess the effects of two prototype HTPs (p-HTPs) on the MucilAir 3D reconstituted human bronchial epithelial cell model compared to the 1R6F reference combustible cigarette. To increase the human-use relevance of the study, cells were exposed to (air) diluted whole smoke/aerosol, and under repeated exposure conditions over 28 days. Effects on cells were assessed throughout the 28-day exposure period using secreted LDH levels as a marker of cytotoxicity, histological evaluation of tissue architecture, goblet cell (Muc5AC) and ciliated cell (FoxJ1) markers, and cilia beat frequency and active area as a measure of cell functionality. To assess the inflammatory responses of the tissues, levels of six markers associated with combustible cigarette smoke exposure were additionally assessed, IL-6, IL-8, MMP-1, MMP-3, MMP-9 and TNFα. This is the first study on the effects of these p-HTPs on the MucilAir (bronchial cell) model, and using a prolonged (28 days), repeat exposure regime.

## Materials and methods

### Test articles

Three test articles were assessed in this study, two prototype heated tobacco products (p-HTPs) (obtained directly from production by Imperial Brands PLC) and the 1R6F Reference Cigarette (Kentucky Tobacco Research and Development Centre, University of Kentucky). The p-HTPs consist of a rechargeable device into which a consumable stick with a reconstituted tobacco portion is inserted (illustrated in Supplementary Figures S1, S2). Upon device activation by the user, the tobacco portion is heated directly with a ceramic heating pin, which generates an aerosol, delivered to the user as they draw air through the filter. The device has two heating temperatures, 315°C and 345°C; the higher temperature of the two was used in this study. Two stick variants were tested, Regular and Intense. The p-HTP sticks were stored at room temperature, protected from light, in sealed portions per test within airtight containers, until use; the 1R6F reference cigarettes were stored frozen, sealed in the original packaging until conditioning according to International Organization for Standardization (ISO, 2018) Guideline 3402 (1999) (at least 48 h at 22 ± 1°C and 60 ± 3% relative humidity) prior to use.

### Cell culture

Fully differentiated reconstituted 3D human bronchial epithelial models (MucilAir™) were purchased from Epithelix Sàrl (Switzerland). The donor was a 41 year-old male Caucasian non-smoker with no pathology (Batch No.: MD072001). Upon receipt, tissues were incubated at 37°C, 5% CO_2_ for 7 days, to acclimatise, according to manufacturer’s instructions. Cells were maintained with 700 μL basal medium (standard manufacturer’s culture medium, Epithelix Sàrl, Switzerland), supplemented with 1% Amphotericin B (Sigma-Aldrich, Germany) (final medium concentration, 2.5 μg/mL). Basal cell culture medium was changed every 3 days, collected in 200 μL aliquots and stored frozen at −80°C until analysis. Once a week mucus was removed from the surface of the tissues: 4 hours following exposure, 200 μL PBS (Mg^2+^/Ca^2+^) was added apically to the tissues and allowed to incubate (37°C, 5% CO_2_) for 30 min, then gently washed from the tissue together with the mucus by manual pipetting. PBS/mucus samples were stored frozen at −80°C for cytotoxicity evaluation (see section, ‘Cytotoxicity Evaluation’).

### Exposures

**FIGURE 1:**
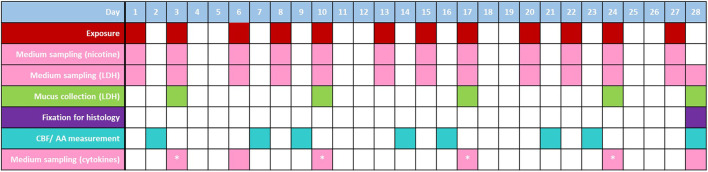
Exposure and sampling/measurement regimes for the 3D models over the experimental period. Samples of medium were taken (following exposures; prior to harvesting on day 28) and mucus (4 h prior to exposures) over the experimental period for LDH release measurement; medium was also sampled for cytokine analysis directly before exposure; on days denoted with *, samples were taken both directly before exposure and 4 h following exposure. Cilia beat frequency (CBF)/active area (AA) measurements were taken on days where exposure did not occur (these measurements were additionally taken on days −5 and 0 to assess tissue functionality prior to the experimental period). Treatments of additionally allocated tissues with 5 ng/mL positive control, IL-13, were also included in the study (days 13, 15, 17, 20, 22 and 24); cells were treated with CBF positive control, procaterol on day 21 (positive controls not included in this figure to increase readability). On day 28, a subset of tissues were fixed for histological analyses.

Whole aerosol/smoke aerosols were applied to the apical surfaces of the 3D models (at the ALI) using the custom-built Smoke Aerosol Exposure *In Vitro* System (SAEIVS) (Rudd et al., 2020; Wieczorek et al., 2020). Exposures were carried out over an experimental period of 28 days (treatment and sampling regimes are detailed in Figure 1). The SAEIVS consists of five smoking chambers into which the test products were placed for respective runs. The 1R6F reference cigarette was smoked according to the ISO 20778 smoking regime (2018) (formerly known as the Health Canada Intense regime) (55 mL puff volume, 2 s puff duration, 30 s puff interval, bell shaped puff profile, ventilation blocking). The p-HTP aerosols were generated using a modified ISO 20778 regime (55 mL puff volume, 2 s puff duration, 30 s puff interval, bell shaped puff profile), with no ventilation blocking. This regime was used for the p-HTPs as there is currently no published ISO regime for HTPs; ventilation blocking was not applied as this is more representative of how the product would be used, i.e., ventilation holes would not be blocked by the user’s fingers or lips as has been suggested with combustible cigarettes (Gee et al., 2018). After each puff, the smoke or aerosol from the five smoking chambers was combined in a mixing pump and diluted with fresh filtered humidified air according to the following ratios (smoke/aerosol in air): 1R6F, 1 in 14 (92.7% dilution; 7.3% smoke concentration); p-HTP Regular, 1 in 2 (50% dilution; 50% aerosol concentration); p-HTP Intense, 1 in 2. Dilutions were applied to prevent excessive toxicity to the cell models (i.e., to achieve sub-cytotoxic exposures) where possible. The diluted aerosol/smoke then moved into exposure chambers where the cell culture plates were placed. Within these chambers (two in parallel) aerosol/smoke is delivered to individual wells *via* a dilution manifold, and a sliding lid allows columns of wells to be sequentially covered to achieve puff-wise exposures across the plate. In this study, 16, 32 or 48 (diluted as described above) puffs were delivered to the cells at each exposure (i.e., equivalent to 1.14, 2.29 and 3.43 puffs for 1R6F; 8, 16 and 24 puffs for p-HTPs Regular and Intense). Following each puff, aerosol/smoke is drawn out of the exposure chamber *via* an exhaust. The SAEIVS achieves delivery of aerosol/smoke generated to cells in <10 s, ensuring that ageing effects are prevented and that maximal aerosol/smoke chemical constituents are delivered to the cells. The numbers of puffs applied were selected to allow for puff count and exposure time comparability between the cigarette smoke and heated tobacco aerosols. This was derived from preliminary dose range finding experiments with the 1R6F reference cigarette smoke (the most toxic test article) (data not shown), where smoke dilutions and puff counts were selected based on non-, sub- and weakly cytotoxic responses in 3D tissues derived from the same donor as used in the main study presented. The pre-study also included selecting the levels of dilution of the fresh 1R6F smoke/p-HTP aerosols with air.

### Nicotine dosimetry

Nicotine dosimetry of the cell culture medium was carried out at the timepoints detailed in Figure 1. Nicotine quantification in medium samples was carried out using LC-MS/MS (AB Sciex API 6500 QTRAP (SCIEX, USA)). For analysis, medium samples were diluted 1:1,000 and 1:2000 with MilliQ water and 1:1 in the autosampler with the internal standard solution in methanol. A Gemini NX-C18 column (110Å, 100 × 2.0 mm, 3 µm) (Phenomenex, USA) was used for the liquid chromatography (oven temperature 55°C ± 1°C), sample injection volume was 5 μL and the autosampler temperature was 5°C. The eluent gradient was applied according to the following: 0min: 2% B (methanol)/98% A (0.05% acetic acid) (flow rate: 400 mL/min); 1.2 min: 65% B/35% A (400 mL/min); 1.5 min: 95% B/5% A (400 mL/min); 2.5 min: 98% B/2% A (400 mL/min); 3.0 min: 98% B/2% A (400 mL/min). The following conditions were used for the mass spectrometry: Ion spray voltage: 450 V, Ion source temperature: 500°C, MRM: 163/132 quantification; 163/106 qualifier. Three replicates were analysed per treatment.

### Cytotoxicity evaluation

Cytotoxicity was assessed based on measurement of levels of LDH release into the cell culture medium and mucus. Medium samples were taken, at the timepoints detailed in Figure 1, prior to exposure, whereas mucus was collected by washing with PBS once a week (see section ‘Cell culture’). For analysis, samples were thawed and analysed using the Promega CytoTox 96^®^ Non-Radioactive Cytotoxicity Assay kit, according to manufacturer instructions. A standard row was used to calculate the total amount of LDH in pg.

### Histology

Tissues from each treatment group, harvested at day 28, were prepared for histological analysis. Tissues were fixed with 3 × 20 min incubations in 4% formaldehyde in PBS (Mg^2+^/Ca^2+^), following this, they were stored in 50 mL tubes filled with PBS at 4°C until processed. Histological analysis was carried out by Epithelix Sàrl. The tissues and culture insert membranes were removed and this disc was cut in half (to be embedded in the same paraffin block). The samples were processed according to the Peloris Automaton (Leica Biosystems, Germany) 1 h protocol, then embedded in paraffin. For each sample, two sections, approximately 3 μm thick, were cut and placed on the same glass slide. Cells were stained with Haematoxylin Eosin (HE)/Alcian Blue (AB) according to Epithelix’s internal protocol. Additional immunohistochemistry staining was carried out on complementary sections using the Ventana Benchmark XT and Ultraview DAB detection kit (760-500) (both Ventana-Roche). Antibodies: staining was carried out for Mucin-5AC protein (Muc5AC) (ThermoFisher Scientific, MA5-12178 (mouse monoclonal)) and forkhead box transcription factor (Fox-J1) (Novus Biological, NBP1-87928 (rabbit polyclonal)). Slides were imaged and digitalised using a Nanozoomer (Hamamatsu) under brightfield conditions, with a ×20 objective (without Z stack mode). Histological quantification was carried out using NDPIExport, developed by Epithelix. For Alcian blue/H&E staining, results were expressed as % positive cells/total area; for Muc5AC, % positive area/total area; and for Fox-J1, % positive nuclei/number of nuclei.

### Cell imaging (CBF, CAA)

Cilia beat frequency (CBF) and cilia active area (CAA) were recorded throughout the experiment, at the timepoints detailed in Figure 1, as an assessment of tissue functionality. The timepoints were selected to fall 24 h pre-/post-exposure to so as not to add any additional stress to the tissues too close to the exposures, and to take the measurements when the tissues are stabilised in the time between exposures. Prior to measurement, tissues were placed into an ibidi Heating System for multiwell plates (ibidi GmbH, Germany) for 20 min at 37°C to acclimatise. Video images of the apical surface of the tissues were captured using the ×4 objective of an Olympus I×53P1F inverted microscope (Olympus, Japan) and analysed using Sisson-Ammons Video Analysis software. The captured image was divided into two blocks (top and bottom), each block was analysed separately using the same routine analysis. Videos were recorded at 200 frames/s (total 1,024 frames/video). Pixel intensities were extracted by the analysis software for a region of interest over time, then data underwent a fast Fourier transformation; noise reduction was applied using a Gaussian distribution. Outputs were displayed as intensity graphs for each of the two endpoints (CBF and CAA).

Procaterol hydrochloride (10 µM final concentration) (Sigma-Aldrich, Germany) was used as a positive control for CBF (increased activity), added to the basolateral medium of dedicated tissues for this analysis. Following addition of procaterol hydrochloride, tissues were allowed 15 min to equilibrate with the compound prior to imaging; the basal medium was removed and exchanged for fresh culture medium after 1 h.

Representative images are included in Supplementary Figure S3.

### Analysis of inflammatory markers

Levels of pro-inflammatory markers tumour necrosis factor alpha (TNFα) and interleukin (IL)-6, the chemokine IL-8 and the matrix metalloproteinases (MMP)-1, MMP-3 and MMP-9 secreted into the basal cell culture medium were assessed at the timepoints detailed in Figure 1. Medium was sampled either directly prior to next exposure (days 3, 6, 10, 17, 24, 28) or 4 h following exposures (days 3, 10, 17, 24). The markers were measured using an MSD^®^ Multi-Spot Assay System MESO Scale QuickPlex™ (MSD Maryland, USA). IL-8 was determined *via* the Chemokine Panel one Kit and a 1:4 dilution of the sampled medium. The other cytokines were measured together on Custom U-Plex plates with undiluted medium samples according to the manufacturer’s protocol.

### Statistical analyses

For the nicotine dosimetry (two-way analysis of variance (ANOVA) with Tukey’s post-hoc test; comparison between numbers of puffs for the respective study products), LDH (two-way ANOVA with Dunnett’s post-hoc test; comparison of the study product responses to Sham for respective timepoints), CBF, CAA (both ordinary one-way ANOVA with Bonferroni’s post-hoc test; comparison of the study product responses to Sham (fold-change) for respective timepoints) and inflammatory marker (two-way ANOVA with Dunnett’s post-hoc test; comparison of the study product responses to Sham (fold-change) for respective timepoints) data, statistical analyses were carried out using GraphPad Prism version 8.

## Results

### Exposure measurement (delivered nicotine)

**FIGURE 2:**
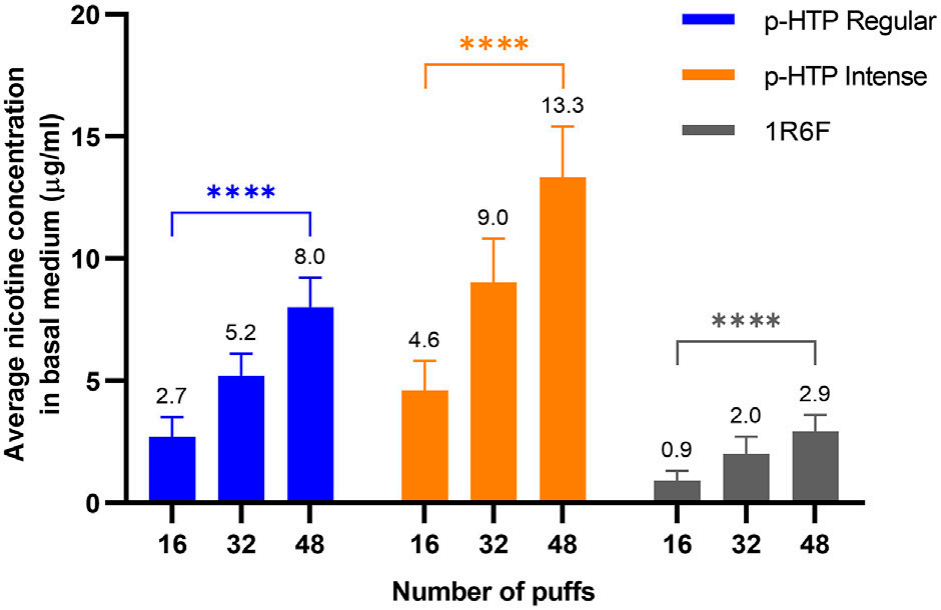
Average (values detailed above each bar) nicotine concentration in basal cell culture medium samples collected directly following exposure of the 3D tissues to 16, 32 or 48 puffs of p-HTP aerosols (diluted 1 in 2) or 1R6F reference cigarette smoke (diluted 1 in 14). Data is an average of measurements in samples taken at the timepoints detailed inFigure 1. Error bars represent standard deviation; *n* = 3 per day x 12 days. Differences between the puff levels of each product were analysed using a two-way ANOVA; ****p < 0.0001 (two-way ANOVA with Tukey’s post-hoc test).

Samples of basal cell culture medium were collected directly following exposure of the models to 16, 32 or 48 puffs of the p-HTP aerosols/1R6F smoke. Nicotine levels within these samples were then quantified to provide an indication of relative exposures to the cultures according to the different exposure levels/products (Figure 2). Measured nicotine levels were approximately proportional to number of puffs delivered for each product, i.e., 16:32:48 puffs = 1:2:3 times the amount of nicotine measured. The p-HTP Intense product variant delivered around 1.7 times more nicotine per puff (diluted 1 in 2) compared to p-HTP Regular, despite the same dilution factor. The lower levels of nicotine observed for 1R6F were due to increased dilution per puff (i.e., 1 in 14) prior to exposures in the cell culture chambers. However, 48 puffs of 1R6F smoke diluted 1 in 14 delivered comparable nicotine levels to 16 puffs of 1 in 2 diluted p-HTP Regular aerosol, indicating that exposures for this study fell within a comparable nicotine delivery range.

### Cytotoxicity

Over the 28-day experimental period, levels of LDH secreted from cells (into basal medium and mucus) were generally consistent between all test articles and Sham at the 16 and 32 puff levels. Although there were a few significant differences to Sham at some timepoints (p-HTP Intense 16 puffs, day 17; 1R6F 16 puffs, day 24 and 32 puffs, days 17 and 22), these were not sustained trends. This was also the case for both p-HTPs with the 48 puff exposures. However, in contrast, increased release of LDH was observed following exposures to 48 puffs of 1R6F smoke compared to Sham, which reached significance at day 10 and generally increased from this point onwards. It was also noted that greater variability was observed for the p-HTP Regular tissues exposed to 32 puffs particularly.

### Histology

**FIGURE 3:**
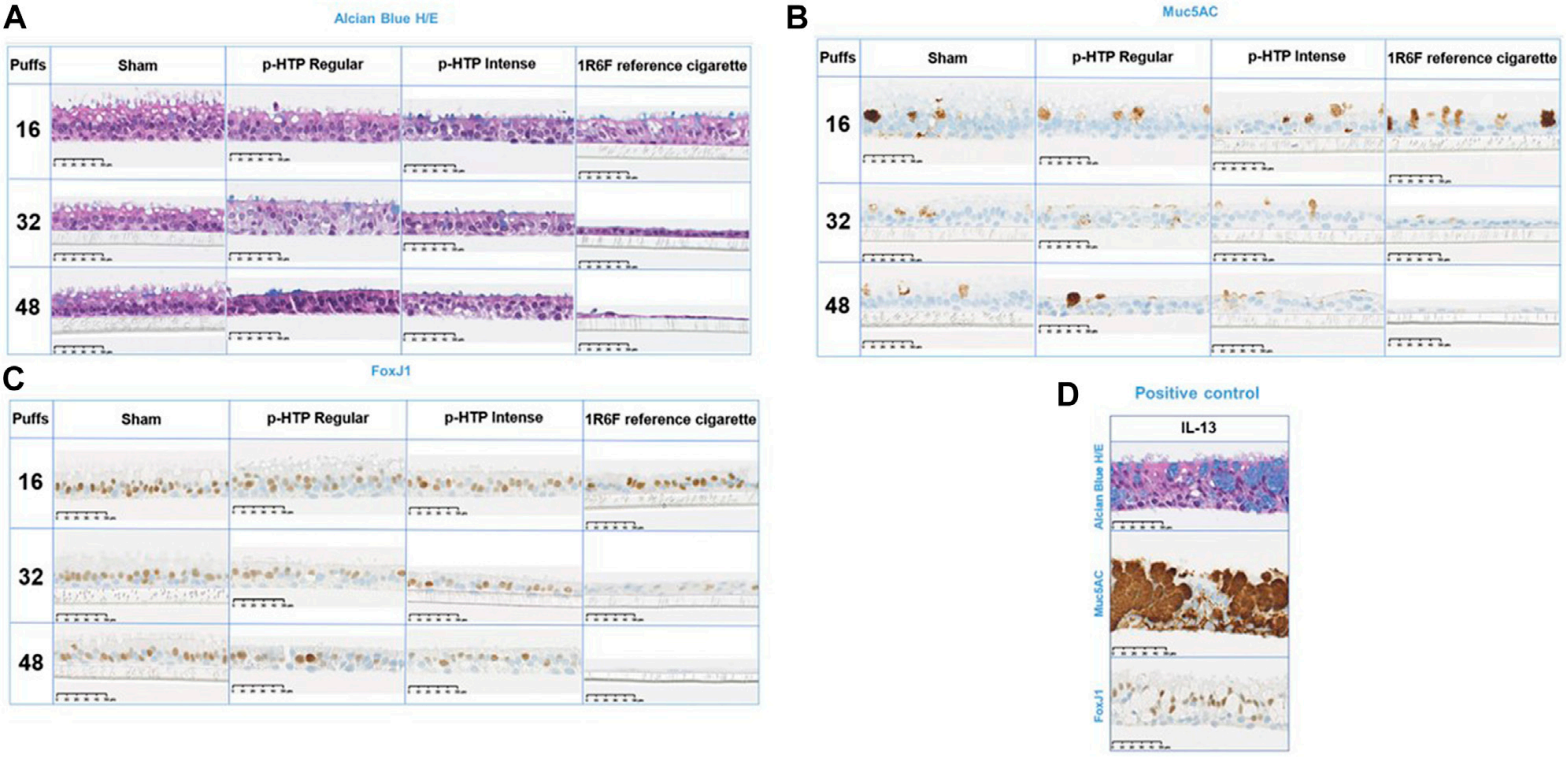
Representative histological images of the tissues exposed to 16, 32 and 48 puffs of air (sham), p-HTP Regular aerosol, p-HTP Intense aerosol or 1R6F reference cigarette smoke, harvested at the end of the 28 days repeated exposure experimental period. Fixed tissue slices were stained with Alcian Blue and H/E **(A)**, Muc5AC **(B)** and FoxJ1 **(C)**. A subset of tissues were treated with IL-13 as a positive control **(D)**. Sham: *n* = 1 per condition; test products: *n* = 2 per condition; IL13: *n* = 3.

Following the 28-day experimental period, a subset of tissues were harvested and fixed for histological analysis (Figure 3). Alcian Blue/H&E staining was carried out to assess tissue architecture, Muc5AC staining was used to indicate the presence of goblet cells and FoxJ1 was used to stain for ciliated cells (Figure 3; Table 1). In the Sham treated tissues, slight declines in all endpoints were observed with increasing numbers of puffs, which may be the result of repeated exposures to puffs, i.e., mechanical stress, over the prolonged 28-day exposure period. For the Alcian Blue/H&E staining, compared to Sham treated tissues, there appeared to be a small decline in tissue height with increasing puffs of the p-HTP aerosols, however, this was not pronounced. For 1R6F, there were clear declines in tissue height and number of cells present, along with changes in morphology with increasing puffs. When stained for Muc5AC, the presence of goblet cells appeared to decrease for all treatments with increasing puffs (including for Sham). However, this effect was especially pronounced for the 1R6F treated tissues with increasing puffs (around 10-fold decrease between 16 and 48 puffs). Puff-wise declines in ciliated cells were observed for the p-HTPs, with slightly stronger effects for p-HTP Intense. However, with increasing puffs of 1R6F smoke, declines in ciliated cells were stronger, with none detected at 48 puffs.

**TABLE 1:** Average staining in treated tissues (16, 32 or 48 puffs of air (Sham), p-HTP Regular, p-HTP Intense, 1R6F), fixed and stained at the end of the 28 days repeated exposure period. Positive control treatment, IL-13, is also included. ND: no staining detected.

		16 puffs	32 puffs	48 puffs
Alcian Blue/H&E (% positive cells/total area)	*Sham*	0.16	0.10	0.10
	*p-HTP Regular*	1.66	0.20	0.03
	*p-HTP Intense*	0.38	0.10	0.08
	*1R6F*	0.25	0.22	0.06
	*IL-13*	19.99		
Muc5AC (% positive area/total area)	*Sham*	4.50	2.97	2.57
	*p-HTP Regular*	5.97	2.07	2.68
	*p-HTP Intense*	3.23	1.75	2.68
	*1R6F*	5.48	1.23	0.54
	*IL-13*	77.26		
FoxJ1 (% positive nuclei/number of nuclei)	*Sham*	47.02	45.93	44.54
	*p-HTP Regular*	45.54	42.17	32.09
	*p-HTP Intense*	42.33	34.10	24.73
	*1R6F*	40.64	17.16	ND
	*IL-13*	43.23		

### Tissue functionality: Cilia active area and cilia beat frequency

**FIGURE 4:**
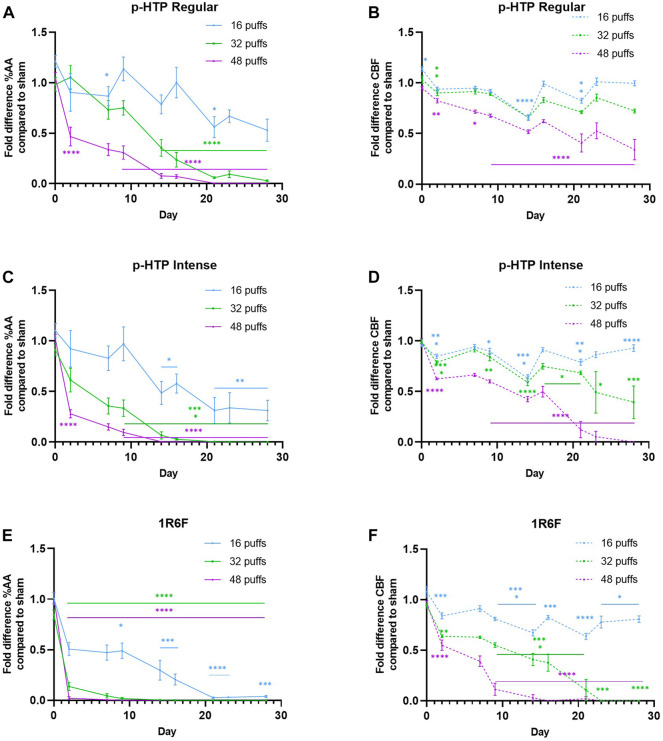
**(A, C, E)** Fold changes in % cilia active area (AA) compared to sham (air) treatment of 3D bronchial tissue models exposed to 16, 32 or 48 puffs of aerosol or smoke **(B, D, E)** Fold changes in cilia beat frequency (CBF) compared to sham (air) treatment of 3D bronchial tissue models exposed to 16, 32 or 48 puffs of aerosol or smoke. Error bars represent standard deviation; Sham: up to day 17, *n* = 5, day 15 onwards, *n* = 3; test products: up to day 17, *n* = 8, day 15 onwards, *n* = 5 (some tissues were lysed for analyses on day 17). *p ≤ 0.05; **p ≤ 0.01; ***p ≤ 0.001; ****p ≤ 0.0001 (ordinary one-way ANOVA with Bonferroni’s *post-hoc* test).

Over the 28 day period, increasing declines in both CAA and CBF compared to Sham (Sham values are detailed in Supplementary Figure S4) over time were observed for all three test articles (Figure 4). The sizes of these declines correlated with the number of puffs to which the tissues were exposed. Additionally, for all test articles, declines in CAA were observed earlier than the declines in CBF. Upon comparison of tissue responses to the two p-HTP aerosols, p-HTP Intense exhibited greater potency than p-HTP Regular, with slightly greater, but not earlier declines in CBF and CAA observed. However, for 1R6F smoke, diluted to a much greater level, declines in CAA and CBF compared to Sham were observed at earlier timepoints and to a much greater degree relative to both the p-HTPs. Some evidence of tissue variability was observed, indicated by differences between Sham and aerosol/smoke-exposed tissues (significant for some p-HTP Regular tissues) at the 0 days timepoint (before any exposures had occurred).

### Inflammatory readouts

**FIGURE 5:**
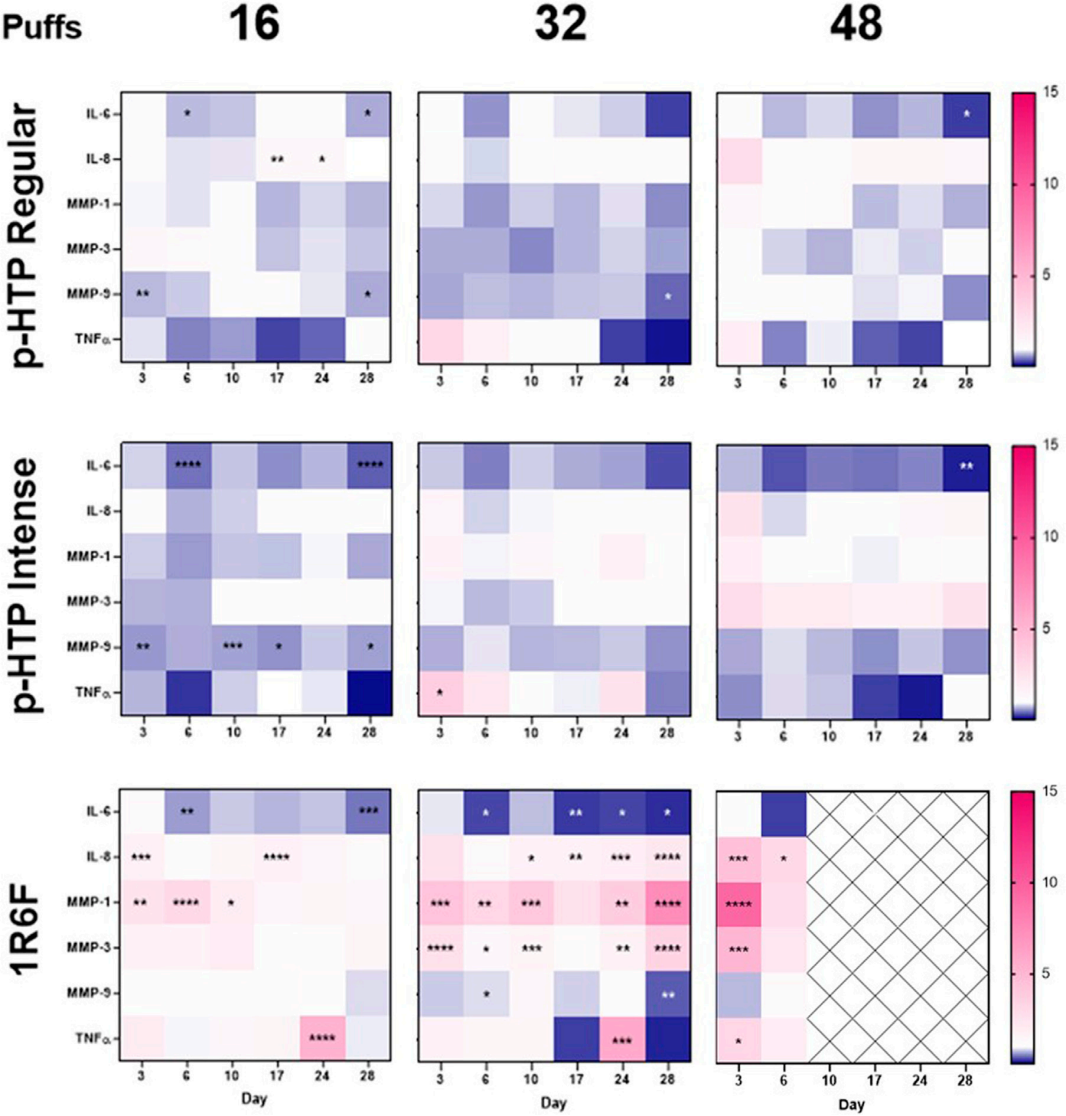
Heatmap representation of inflammatory markers in the cell culture medium of models exposed to 16, 32 or 48 puffs of p-HTP Regular or Intense aerosol or 1R6F smoke. Cells were exposed according to the regime detailed in Figure 1 and levels in basal medium samples taken pre-exposure on days 3, 6, 10, 17, 24 and 28 are shown. Data is plotted as fold-change relative to Sham (1-fold); the highest value on the heatmap is set to the highest observed fold-change across the six markers measured (15-fold) and the lowest value is set to the equivalent inverse. Pink indicates an increase in secretion of markers into the medium compared to Sham levels, and blue indicates a decrease; crossed out cells indicate timepoints where significant cytotoxicity was first observed for 1R6F. Statistically significant changes from Sham levels are denoted by asterisks (*): *p ≤ 0.05; **p ≤ 0.005; ***p ≤ 0.001; ****p ≤ 0.0001 (two-way ANOVA with Dunnett’s post-hoc test). Data plots can also be found in the supplementary information (Supplementary Figures S5–S10), along with measurements from samples taken 4 h post-exposure. *n* = 3.

**FIGURE 6:**
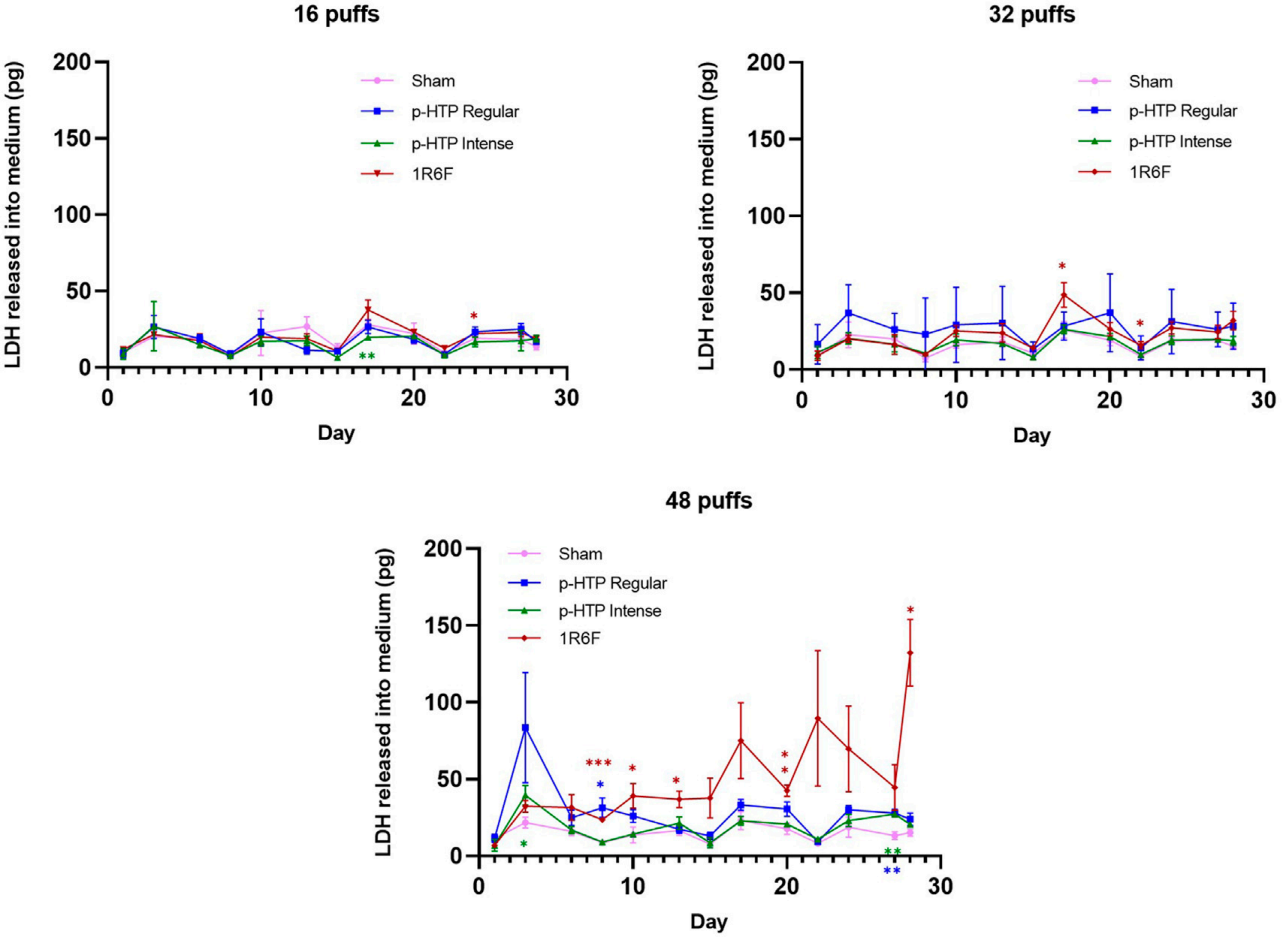
LDH released into cell culture medium and mucus from tissues exposed to 16, 32 and 48 puffs of sham (air), p-HTP Regular, p-HTP Intense and 1R6F Reference Cigarette. For each puff level, responses for p-HTP Regular, p-HTP Intense and 1R6F were statistically compared to sham measurements for each day measured (1, 3, 6, 8, 10, 13, 15, 17, 20, 22, 24, 27, 28); *p ≤ 0.05, **p ≤ 0.005, ***p ≤ 0.001 (two-way ANOVA with Dunnett’s post-hoc test). Error bars represent standard deviation; *n* = 3.

Measurement of the levels of six inflammatory markers secreted into the basal cell culture medium was carried out; samples were taken either directly before exposure (Figure 5; Supplementary Figures S3–S8) or 4 h post-exposure (Supplementary Figures S6–S9). For IL-6 and TNFα, no signal was detected in the samples post-exposure, and therefore data is not shown. Although there were few significant changes in TNFα levels across the timepoints measured (p-HTP Intense on day 3, 32 puffs; 1R6F on days 3 (48 puffs) and 24 (16 and 32 puffs)), there were some initially elevated levels on day 3. This response was similar for 1R6F, however, on day 24 there was a large peak in response for 16 and 32 puffs. The IL-6 response measured following one exposure (measured on day 3) to the test articles did not significantly differ to Sham. However, a second exposure led to declines in levels, which was significant for both p-HTPs for 16 puffs only and for 1R6F at 16 and 32 puffs only (Figure 5; Supplementary Figure S3). For the p-HTPs, levels of IL-6 did not significantly differ to Sham again until the measurement on day 28, and this was for 16 and 48 puffs only. In slight contrast, significant decreases were observed for 1R6F from day 17 onwards, however, for 48 puffs, on day 28, there was a sharp increase in levels secreted into the medium. The strongest IL-8 responses were induced by exposure to 1R6F smoke in both the pre- and post-exposure samples, with both immediate and sustained elevations in levels secreted. At the second post-exposure timepoint (day 10), there were significant elevations in the levels of IL-8 at all three puff levels for the p-HTPs, however, there were no significant changes relative to Sham in the pre-exposure samples. These responses were reflected in the levels of MMP-1 secreted, in both the pre- and post-exposure samples. For both IL-8 and MMP-1, effects were clearly puff dependent, particularly for 1R6F, which elicited substantially greater responses than the p-HTPs. MMP-3 and MMP-9 responses were generally less pronounced, with little trend in significant deviations from Sham for MMP-9. However, MMP-3 secretion was significantly increased for 1R6F exposures at 32 and 48 puffs in the pre-exposure samples. Post-exposure at the earlier timepoints and higher puffs, there were some significant increases in MMP-3 secretion, however, in contrast, p-HTP Regular induced increasing secretions post-exposure with time at the 32 puffs level only, which was coupled with high variability between replicates. However, as 48 puffs of 1R6F induced significant cytotoxicity from day 10 until the end of the experimental period (Figure 6), this data was omitted from further interpretation with regards to inflammatory responses, as these responses would likely be associated with cell death rather than progression to disease-representative pathologies (Iskandar et al., 2018).

## Discussion

This study was the first to assess the effects of the two p-HTP variants’ whole aerosols on the 3D MucilAir model. Furthermore, this study was carried out over an extended (28 days) period, using repeat exposures, and therefore more representative of a realistic adult smoker exposure scenario.

### Exposure to p-HTP aerosols resulted in substantially reduced toxicological outcomes compared to 1R6F smoke

The 3D tissues were exposed to 16, 32 or 48 puffs of diluted p-HTP aerosol (1 in 2) or 1R6F smoke (1 in 14) and across the endpoints assessed, both p-HTP variants were substantially less toxic than 1R6F. Upon assessment of LDH secretion into both the basal cell culture medium and mucus across the experimental period, at 16 and 32 puffs, the LDH levels released by tissues exposed to the p-HTPs did not significantly differ when compared to Sham across the timepoints measured. There was increased variability in tissue responses to p-HTP Regular exposures (particularly for 32 puffs), which was also observed across the experimental endpoints assessed within this study, and therefore may be due to comparatively increased sensitivity of certain tissues. Tissues repeatedly exposed to 48 puffs of 1R6F smoke demonstrated the greatest increase in LDH secretion, which generally increased with time, and furthermore, the dilution level of smoke was one in 14, much greater than for the p-HTP aerosols (1 in 2). With the exception of the high cytotoxicity induced by 1R6F at 48 puffs from day 10 onwards, exposures delivered were generally sub-cytotoxic and therefore responses observed in the subsequent endpoints assessed were deemed to be related to processes potentially leading to disease pathologies rather than cell death.

Upon histological analysis of the tissues, slight declines in staining were observed with increasing puffs for the Sham treatment, which mirrors the secretion of LDH by the Sham tissues over the experimental period (Figure 6). However, as tissues were fixed for histological analysis at the end of the experimental period, they may have undergone some effects from mechanical stress of puffing with air over a long-term period. Declines in function in both incubator and air control MucilAir tissues following an extended experimental period were also observed by Haswell et al. (2021) and following a single non-cytotoxic exposure (Phillips et al., 2021). It must be acknowledged that these models cannot fully replicate a human *in vivo* scenario, where interplay of other cell types, in addition to being part of a whole tissue, as well as mechanical stimulation *in situ*, may have an effect on cellular and tissue maintenance.

However, upon comparison of Sham tissues to both the p-HTP aerosols and 1R6F smoke, there were clear differences between the responses observed. 1R6F induced increasing declines in normal tissue morphology (Alcian Blue/H&E staining) and the greatest loss of goblet (Muc5AC staining) and ciliated (Fox-J1 staining) cells with increasing numbers of puffs. Changes followed a similar trend for the p-HTPs, but these were more subtle and more closely resembled Sham responses. Some measures were relatively elevated for certain staining endpoints for p-HTP Regular (16 puffs, Alcian Blue/H&E and Muc5AC), which may be attributed to tissue variability, as observed across the other study assays for this product variant. However, p-HTP Intense generally demonstrated the higher potency of the two variants in the histological analyses (discussed further in ‘*Nicotine dosimetry and translation to exposure to other chemicals’* section). In the study conducted by Haswell et al. (2021), sub-cytotoxic, and much higher dilutions, of 1R6F smoke (but not HTP aerosol) induced goblet cell hyperplasia within a 6 weeks experimental period. Although the current study did not cover as long a time period, and applied higher exposures of smoke, we only observed some decline in goblet cells. This indicates that both exposure dose and duration are potentially important factors in the experimental design.

Changes in CAA and CBF are recognised indicators of airway epithelial dysfunction (Gindele et al., 2020; Luettich et al., 2021); Luettich et al. (2021) recently described their role in the AOP to decreased lung functionality upon exposure to inhaled toxicants. In this study, CBF and CAA were recorded at timepoints across the 28 day period to understand changes in functionality in response to the repeat exposure regimens. Across the experimental timepoints (particularly at the lower puffs levels for the p-HTPs) variation in the levels of change compared to Sham were observed. This could be due to some capacity of cells/tissues to recover between exposures. However, pronounced declines in CAA were followed by those in CBF and the effects on the presence and functionality of cilia correspond to the histological outcomes. Overall, it was clear that the effects of 1R6F smoke (diluted to a higher level) were substantially greater, and generally occurred earlier, than those of the p-HTPs. Generally greater effects of combustible cigarette smoke, compared to other test articles, in repeated exposure studies with such models have also been previously observed (Czekala et al., 2021; Haswell et al., 2021).

Inflammation is another key process in airway damage and dysfunction and the associations of inflammatory mediators with exposure to combustible cigarette smoke have been extensively mapped (Aghapour et al., 2018). Many *in vitro* studies into NGP aerosol/cigarette smoke exposures have included inflammatory cytokine panels as an indicator of cellular/tissue responses to exposure (Iskandar et al., 2019; Czekala et al., 2021; Phillips et al., 2021). Here, we selected six markers associated with combustible cigarette smoking to assess how responses compared between the p-HTP aerosols and 1R6F smoke. As observed with CAA/CBF, across the experimental period, there was some variation in levels of responses compared to Sham dependent on the timepoint, which again, could be due to some adaptive responses of the tissues. Across all of the selected markers, 1R6F induced the strongest and most sustained responses, which were substantially greater than those to the p-HTPs. Due to the significant and increasing cytotoxicity from day 10 onwards for 48 puffs of diluted 1R6F smoke, the readouts were regarded to be due to pathways related to cellular death rather than to a disease pathological state, as described by Iskandar et al. (2018).

The six inflammatory markers assessed within this study have all demonstrated associations with exposure to cigarette smoke, and particularly associated with disease pathologies including the chronic inflammatory condition, COPD (Zhang et al., 2011; Tanaka et al., 2014; Ostridge et al., 2016; Kraen et al., 2019; Huang et al., 2021). Four hours following the exposures, across the experimental days, IL-6 and TNFα were not consistently detectable in the medium for any of the treatments (including Sham), which may be due to the sensitivity of the method coupled with low, if any, secretion by that sampling timepoint. Therefore, data is not shown. IL-6 secretion was generally supressed compared to Sham following exposures to all three test articles, however, this effect was generally not significant for the p-HTPs (with the exception of 16 puffs on day 6 and 16 and 48 puffs on day 28). Pathways involving c-jun and NFκB have an association with IL-6 suppression (Tanaka et al., 2014), and in the previous study by Chapman et al. (2023), both the p-HTP Regular product and 1R6F induced significant (dose dependent) increases in c-jun and NFκB activity, albeit to different degrees (and in a single human bronchial cell type model and measured at two timepoints only). Iskandar et al. (2018) also observed decreases in IL-6 upon single exposure of tissues to 1R6F, however, not following exposure to HTP aerosol. The increase in IL-6 secretion at day 28 for cells exposed to 1R6F may be the artefact of high cell death by this timepoint for this exposure.

Following an initial non-significant increase in IL-8, particularly at 48 puffs, for the p-HTPs, limited changes in the pre-exposure collected medium suggested that there was limited to no sustained response over the experimental period. However, for 1R6F, IL-8 levels remained significantly elevated across the experimental period for 48 puffs, with a sharp increase in secretion between days 10 and 24, followed by a decline, correlating with the observed cytotoxicity for this treatment. Tissues exposed to 32 puffs of 1R6F exhibited consistently significantly elevated levels of IL-8 secretion present in the pre-exposure medium samples, however to a lesser degree than observed with the cytotoxic 48 puffs. This trend was also observed, but again to a slightly lesser extent, for 16 puffs of 1R6F. For the p-HTPs, secretions measured 4 h post-exposure indicated some initial responses to exposure up to day 10, however, for the two timepoints measured following this (days 17 and 24), there were no significant changes, potentially indicating an adaptive response of the tissues. In contrast, exposures to all puff levels of 1R6F smoke induced secretion of IL-8 following exposure. Interestingly, although IL-8 (and TNFα) has been implicated in the post-transcriptional regulation of MUC5AC gene expression (Bautista et al., 2009), we observed puff-dependent declines in MUC5AC staining across all treatments.

Upon assessment of MMP-9, despite some significant changes compared to Sham, there was no clear trend in responses, which were weak compared to those seen for the other two MMPs, particularly in response to 1R6F exposure. For the p-HTPs, there were few and small significant changes compared to Sham for MMP-3, with the exception of at day 24 for 16 puffs, 4 h post-exposure for p-HTP Regular. However, this was coupled with high variability, as seen in the other endpoints for this set of tissues, and therefore it is unclear how reliable this particular response is. Despite some apparent adaption of tissues to exposure to 1R6F at day 17, and increase in MMP-3 was observed at the later timepoints, again correlating with the cytotoxicity/cell loss observed towards the end of the experimental period. On day 10, in the medium sampling following exposure to the p-HTPs, the tissues demonstrated significant increases in MMP-1 secretion at all three puff levels; however, as observed with the other inflammatory markers, this was followed by subsequent apparent adaption of the tissues to this treatment. In contrast, 1R6F elicited significant increases in secretion of MMP-1 from the first measurement of the experimental period, and these responses to 1R6F were particularly sustained and apparent throughout the experimental period. MMPs have a number of roles within the inflammatory response, for example in regulation of cytokines and chemokines and in tissue remodelling (Czekala et al., 2021), however, their activity and exact roles in the human lung is complex (Churg et al., 2012). A lower MMP-9 response in such tissues compared to MMP-1 and MMP-3 to combustible cigarette exposure was also observed by Czekala et al. (2021).

Although broadly non-significant, TNFα levels were variable compared to Sham, and were particularly elevated at the first measured timepoint (day 3), potentially due to an initial stress response. However, lower levels of TNFα are involved in various cellular/tissue processes under homeostatic conditions (Mukhopadhyay et al., 2006), and therefore the lowered TNFα levels observed here may correlate with the adaptive responses observed in tissues, particularly those exposed to the p-HTPs. At day 24, there was a peak in TNFα secretion for 16 and 32 puffs of 1R6F, which may signal a heightened stress response in the tissues following cumulative exposures. However as TNFα is known to be involved in many cellular processes, including driving inflammatory responses and also apoptosis (Mukhopadhyay et al., 2006; Malaviya et al., 2017), the readouts here require further investigation, as is true for all the markers assessed. Furthermore, inflammatory responses are complex and involve the interaction of many molecules and pathways, therefore further resolution on the effects of the test articles on these would be the focus of future studies, for example through transcriptomics analyses.

Overall, the *in vitro* assessment findings support the observations in other studies with 3D models exposed to combustible cigarette smoke and HTP aerosols, that effects are substantially greater upon exposure to cigarette at lower concentrations (Iskandar et al., 2017; Iskandar et al., 2018; Haswell et al., 2021). However, further studies are required on the effects of HTPs following repeated exposures to substantiate the observations from this study.

### Whole aerosol/smoke repeated exposures to 3D human cell models increase human relevance of the outcomes

This study utilised the MucilAir 3D reconstituted human bronchial epithelial cell model, an established and robust model which has been utilised in a number of inhalation toxicological assessments (Huang et al., 2013; Frieke Kuper et al., 2015; Bedford et al., 2022). The use of human-derived cells increases the relevance of the outputs to consumers and can more closely represent human-specific molecular pathways and responses (Krewski et al., 2010; Adeleye et al., 2015). Furthermore, the repeated exposure element of the study more accurately models likely human exposure patterns than a single, acute, *in vitro* exposure (Mallock et al., 2019; Jones et al., 2020; Laverty et al., 2021). Interestingly, lower or absent responses in 3D respiratory tissue models exposed to heated tobacco aerosol, compared to cigarette smoke, in the short term (e.g., up to 72 h), following an acute exposure (Iskandar et al., 2018; Mori et al., 2021) was a relative response observed to be maintained over the 28 days of the current study. The findings of the current study therefore indicate reduced harm potential of the p-HTPs over the longer period tested. However, the 28 days exposures applied within this study still provide valuable insight into outcomes upon repeated exposures over a prolonged period for both the p-HTPs and cigarette.

In combination with this, whole aerosol/smoke exposures were achieved using the SAEIVS, which enables a number of human-relevant conditions to be met during exposure. Firstly, the whole aerosol/smoke exposures allow the delivery of all chemical fractions generated upon heating/combustion (respectively) of the products, including particulate and gas/vapour phases; delivery of a combination of these fractions is often challenging in *vitro* systems, for example, in submerged cell cultures, as evaluated previously (Smart and Phillips, 2021). In addition to the delivery of all chemical fractions, the SAEIVS ensures exposures of cells to aerosol/smoke within 10 s of generation, preventing ageing effects. These, in combination, ensure that cells are exposed to as consumer-relevant chemical mixture as possible. Further to this, humidity within the SAEIVS is maintained at 70%–80%, to prevent tissue drying effects throughout the exposures. A vacuum pump also acts to remove aerosol/smoke from the exposure chambers following each puff, mimicking exhalation of some chemicals.

### Nicotine dosimetry and translation to exposure to other chemicals

Nicotine is often used as a marker of aerosol/smoke exposure *in vitro* as it is considered to be a reliable dosimetry measure (Adamson et al., 2016; Behrsing et al., 2018). In this study, nicotine dosimetry using the levels trapped within the basal medium was carried out to gain an understanding of relative exposures to the tissues. During exposure, gaps present at the sides of the culture inserts for gaseous exchange in the basal medium of the system enable delivery to and trapping of the aerosol/smoke constituents in this compartment. Although this does not model deposition on the tissue surface, it does provide an indication of relative exposures and has previously been used as a dosimetry method in similar 3D culture exposure set-ups (Haswell et al., 2017).

Although exposures were matched on a puff basis, matching delivered nicotine levels across all exposure levels was not possible in combination with this due to the high cytotoxicity potential of 1R6F smoke at lower dilutions. However, average nicotine levels delivered by 16 puffs of the p-HTP Regular product did match those delivered by 48 puffs of 1R6F, allowing some comparison of effects upon a nicotine basis. Overall, it is clear that 1R6F smoke induced substantially greater toxicological effects compared to the p-HTPs, across a comparable nicotine delivery range. Although adult smokers smoke for nicotine amongst other reasons, tobacco combustion generates more than 7,000 chemicals (US Department of Health and Human Services, 2014), to which the consumer is exposed. A number of studies have demonstrated, however, that aerosols generated from HTPs are less complex and contain fewer and substantially lower levels of toxicants compared to combustible cigarette smoke, attributed to the heating, as opposed to burning of tobacco (Jaccard et al., 2017; Forster et al., 2018; Malt et al., 2022). A recent study by Chapman et al. (2023) characterised the aerosols generated by two p-HTPs, one of which was used in this study (p-HTP Regular) and reported substantial reductions in the levels and numbers of toxicants present in the aerosol compared to 1R6F smoke. On a per puff basis, nicotine delivery was around half that for the p-HTPs compared to 1R6F, however, on a nicotine equivalent basis, substantial reductions in *vitro* toxicological outcomes were observed upon exposure of cells to the p-HTP aerosols. To maximise adult smoker satisfaction, the p-HTP Intense variant used in this study was designed to deliver increased aerosol, and therefore nicotine per puff, and indeed greater levels of nicotine were measured in the basal medium following exposures. In the case that correspondingly increased toxicant levels were delivered to the tissues, in line with the HT product category, these levels are still expected to be substantially reduced compared to (1R6F) combustible cigarette smoke. Indeed, toxicological responses observed were similar between the two p-HTP variants used in this study, supporting the growing evidence for the THR potential of this category (Committee On Toxicity, 2017; Mallock et al., 2018; McNeill et al., 2018; RIVM, 2018; SHC, 2020). *In vitro* analyses with more mechanistic resolution, for example, transcriptomics approaches, may provide more resolution between toxicological responses to individual variants within the same product category, and will be the subject of future studies.

Although aerosol/smoke deposition on the tissue surfaces was not measured, it would be informative to gain an understanding of aerosol/smoke deposition and therefore apical exposures to the tissues. Additionally, this information, in combination aerosol particle size data, could be inserted into lung deposition models to predict human relevant exposure scenarios. This will also be addressed in future studies. Furthermore, the basal nicotine concentrations measured within this study were markedly higher than physiological blood plasma nicotine levels in smokers or heated tobacco product users (10–50 ng/mL, Benowitz et al., 2009; Phillips-Waller et al., 2021). Whilst not at physiological levels, the quantification of nicotine was useful in providing an indication of relative exposures to the cultures according to the different exposure levels/products.

### The role of NGPs in THR

This study has demonstrated that the human 3D bronchial epithelial cell models exhibit substantially reduced toxicological responses following exposure to the p-HTP aerosols compared to 1R6F reference cigarette smoke. This is consistent with the current scientific evidence base underpinning the HTP category and reflects the previously described substantial reductions in the levels and numbers of toxicants present in the aerosols of p-HTPs compared to 1R6F smoke correlating with reduced toxicological outcomes observed across a range of *in vitro* models (Chapman et al., 2023). As may be expected, although the p-HTPs do not exhibit the same level of reduction in toxicological responses as typically observed with ENDS (Czekala et al., 2021), the data in this manuscript does supports the proposed placement of nicotine delivery products across a relative risk scale (Abrams et al., 2018; Murkett et al., 2020). However, HTPs, as demonstrated in this study, still offer substantially reduced harm nicotine delivery compared to combustible cigarette smoking (Committee On Toxicity, 2017). Further to this, for NGPs to reach their full THR potential, they must offer adult smokers an acceptable form of nicotine delivery, which includes sensory satisfaction, which HTPs may provide to adult smokers as a closer experience to cigarette smoking (Roulet et al., 2019; Haziza et al., 2020).

### Limitations of the study and future directions

This study provides valuable information on the effects of repeated exposures, over a prolonged period of 28 days, to p-HTP aerosols in the MucilAir bronchial cell model compared to combustible cigarette smoke. The study does have a number of limitations which must be acknowledged, and the data viewed within this context. Some variability between tissues was observed, particularly for the subset of models used for the p-HTP Regular exposures, which may remove some resolution of the subtle differences in effects of the two product variants. Whilst technical replication was carried out to mediate such biological variability, and tissues underwent quality control measures prior to the start of experimentation (transepithelial electrical resistance (TEER) measurement/visual (microscopic) inspection), the study was conducted using models derived from one donor, and therefore does not account for donor variability. This is an important consideration as, for example, in the study by Czekala et al. (2021), tissues were exposed to greater numbers of reference cigarette puffs, which although diluted slightly more than in the current study (1:17 vs 1:14 respectively), tissues appeared more sensitive to combustible cigarette smoke in the present study, and puffs were limited to 48 to prevent excessive cytotoxicity. However, it is difficult to fully model consumer variability *in vitro*, and the recent findings of Bowers et al. (2021) indicate that 13–299 donors would be required to provide information on various inflammatory readouts. Additionally, the current study was carried out on models derived from human bronchial cells, however, this does not model other regions of the respiratory tract, for example the alveoli, and does indicate the interaction with other cell types present *in vivo*, including fibroblasts, endothelium and immune cells. Assessment of the effects of the test products in models including alveolar tissues and those in the presence of immune cells will be the focus of future work.

To further expand upon the mechanistic insights into the effects of the test products, it would also be beneficial to expand upon the endpoints assessed in the current study to include analyses at the transcriptomic level, to identify further pathways involved in the responses, and gain information on important processes to screen for in future *in vitro* assessments. For example, the 3D models demonstrate metabolic capability (Huang et al., 2013; Cervena et al., 2019) and whilst no measurements of this activity were taken in the current study, it would be interesting to gain some insight into metabolic activity and upregulation upon exposure to the respective test articles. From this capacity of the tissues, however, it could be assumed that exposure outcomes within the current study may have involved the effects of the metabolism pro-toxicants, and warrants further investigation.

The experimental design would also benefit, in future studies of this type, from switching/dual exposure arms. These are two important use scenarios to model *in vitro*, and would further increase the consumer relevance of such studies: NGPs offer adult smokers an alternative, reduced harm form of nicotine delivery to adult smokers, therefore switching from cigarette use to NGP use could be modelled by a period of exposure to combustible cigarette smoke followed by a period of exposure to NGP. Switching is often accompanied by a period of dual use of combustible cigarettes and NGPs (Farsalinos et al., 2015; Adriaens et al., 2017; Sutanto et al., 2020), therefore, an *in vitro* model of this may also be informative. However, how *in vitro* responses translate to *in vivo* effects still remains to be elucidated.

Finally, in future studies it would beneficial to extend endpoints to add to the weight of evidence with regards to the outcomes. For example, transepithelial electrical resistance (TEER) measurement can provide an additional measure for barrier integrity and will be incorporated as an endpoint in future studies of this kind.

## Conclusion

This study has demonstrated that the effects of repeated exposures of 3D human reconstituted bronchial cell models *in vitro*, over an extended 28 day period, to p-HTP variant aerosols are substantially reduced compared to the effects of 1R6F combustible reference cigarette smoke. This is in line with the findings of previous *in vitro* studies on both the p-HTPs and HTP category, and supports a growing weight of evidence for the tobacco harm reduction potential of such products, through offering a potentially less harmful form of nicotine delivery to adult smokers. The combination of the human MucilAir models, whole aerosol/smoke exposures and an extended, repeated exposure regime additionally increases the human, and therefore consumer, relevance of the outcomes observed.

## Data Availability

The original contributions presented in the study are included in the article/supplementary material, further inquiries can be directed to the corresponding author.

## Abbreviations

ALI, air-liquid interface; COPD, chronic obstructive pulmonary disease; HPHCs, Hharmful and potentially harmful constituents; HTP, heated tobacco product; ISO, international organization for standardization; NGP, next-generation product; p-HTP, prototype heated tobacco product; SAEIVS, smoke aerosol exposure *in vitro* system; THR, tobacco harm reduction.
