# Supplementary material for: Twenty-eight day repeated exposure of human 3D bronchial epithelial model to heated tobacco aerosols indicates decreased toxicological responses compared to cigarette smoke
Source: Front Toxicol. 2023 Feb 16;5:1076752. doi: 10.3389/ftox.2023.1076752 (PMC9979258; doi:10.3389/ftox.2023.1076752)
Supplement: Supplementary file 1 [file DataSheet1.docx]

**Twenty-eight day repeated exposure of human 3D bronchial epithelial model to heated tobacco aerosols indicates decreased toxicological responses compared to cigarette smoke**

Fiona Chapman^1^, Sarah Jean Pour^2^, Roman Wieczorek^2^, Edgar Trelles Sticken^2^, Karin Röwer^2^, Sandra Otte^2^, Elizabeth Mason^1^, Lukasz Czekala^1^, Thomas Nahde^2^, Grant O’Connell^1^, Liam Simms^1^, Matthew Stevenson^1^

^1^ Imperial Brands PLC, 121 Winterstoke Road, BS3 2LL, Bristol, UK

^2^ Reemtsma Cigarettenfabriken GmbH, Albert-Einstein-Ring-7, D-22761, Hamburg, Germany

**Supplementary information**


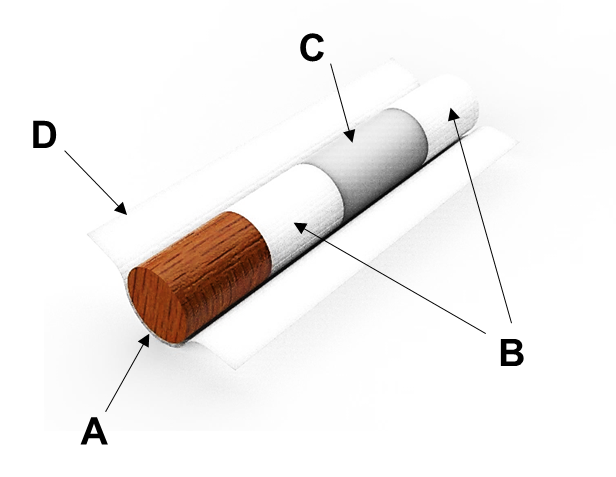


**Figure S1**: Representative diagram of the p-HTP sticks used in this study, consisting of reconstituted tobacco (**A**), filters (**B**), a cardboard tube (**C**) and outer paper (**D**).

**
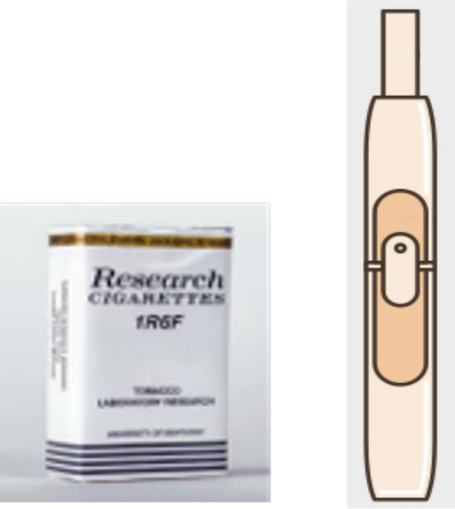
**

**Figure S2**: Representative diagram of the p-HTP device used in this study (reconstituted tobacco stick inserted).

**
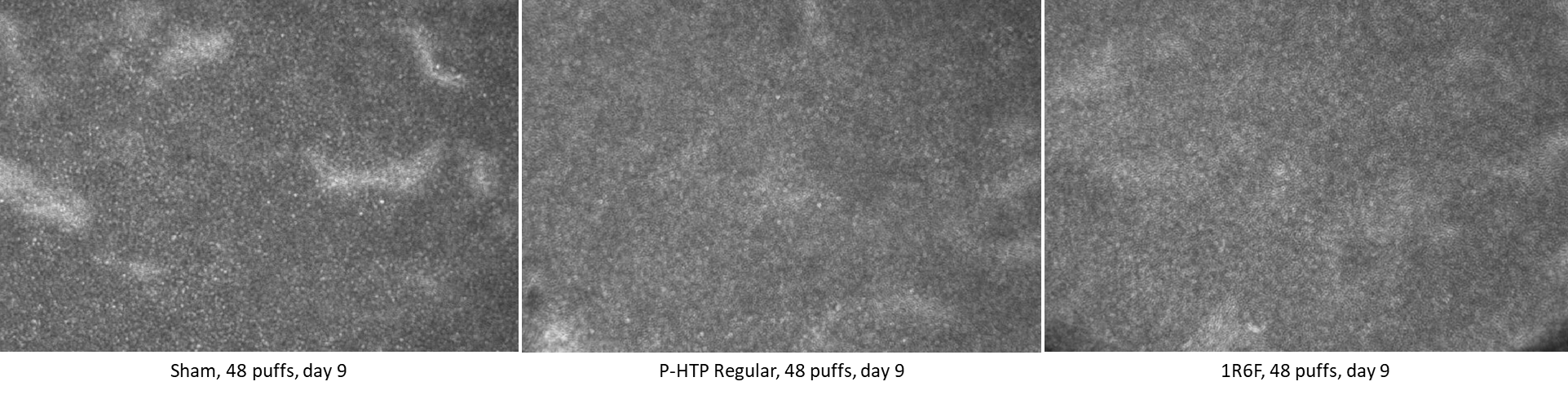
**

**Figure S3**: Still images from the analyses carried out for cilia active area and beat frequency within the study. Images are from the 9 day timepoint, and are of tissues exposed to 48 puffs of air (Sham), p-HTP (Regular) and 1R6F reference cigarette.

**Figure S4**: Sham treatment cilia active area (CAA) (%) and beat frequency (CBF) () across the 28 day exposure period. These values were used to calculate the fold changes in responses compared to Sham in Figure 5. A mixed effects analysis was carried out to compare all timepoints’ mean values to T0 for the respective numbers of puffs applied per exposure (16, 32, 48 puffs) and significant changes are denoted by asterisks (*): *p<0.05.

**Figure S5**: Mean fold-change levels compared to Sham of IL-6 measured in the basal cell culture medium sampled prior to next exposure (day 28, prior to harvesting) of tissues to 16, 32 or 48 puffs of p-HTP Regular, p-HTP Intense aerosols or 1R6F smoke. Error bars represent standard deviation; n=3. Statistically significant changes from Sham for each timepoint are denoted by asterisks (*): *p≤0.05; **p≤0.005; ***p≤0.001; ****p≤0.0001 (two-way ANOVA with Dunnett’s post-hoc test).

**Figure S6**: Mean fold-change levels compared to Sham of IL-8 measured in the basal cell culture medium sampled prior to next exposure (day 28, prior to harvesting) (a, b, c), or 4h following exposure (d, e, f), of tissues to 16, 32 or 48 puffs of p-HTP Regular, p-HTP Intense aerosols or 1R6F smoke. Error bars represent standard deviation; n=3. Statistically significant changes from Sham for each timepoint are denoted by asterisks (*): *p≤0.05; **p≤0.005; ***p≤0.001; ****p≤0.0001 (two-way ANOVA with Dunnett’s post-hoc test).

**Figure S7**: Mean fold-change levels compared to Sham of MMP-1 measured in the basal cell culture medium sampled prior to next exposure (day 28, prior to harvesting) (a, b, c), or 4h following exposure (d, e, f), of tissues to 16, 32 or 48 puffs of p-HTP Regular, p-HTP Intense aerosols or 1R6F smoke. Error bars represent standard deviation; n=3. Statistically significant changes from Sham for each timepoint are denoted by asterisks (*): *p≤0.05; **p≤0.005; ***p≤0.001; ****p≤0.0001 (two-way ANOVA with Dunnett’s post-hoc test).

**Figure S8**: Mean fold-change levels compared to Sham of MMP-3 measured in the basal cell culture medium sampled prior to next exposure (day 28, prior to harvesting) (a, b, c), or 4h following exposure (d, e, f), of tissues to 16, 32 or 48 puffs of p-HTP Regular, p-HTP Intense aerosols or 1R6F smoke. Error bars represent standard deviation; n=3. Statistically significant changes from Sham for each timepoint are denoted by asterisks (*): *p≤0.05; **p≤0.005; ***p≤0.001; ****p≤0.0001 (two-way ANOVA with Dunnett’s post-hoc test).

**Figure S9**: Mean fold-change levels compared to Sham of MMP-9 measured in the basal cell culture medium sampled prior to next exposure (day 28, prior to harvesting) (a, b, c), or 4h following exposure (d, e, f), of tissues to 16, 32 or 48 puffs of p-HTP Regular, p-HTP Intense aerosols or 1R6F smoke. Error bars represent standard deviation; n=3. Statistically significant changes from Sham for each timepoint are denoted by asterisks (*): *p≤0.05; **p≤0.005; ***p≤0.001; ****p≤0.0001 (two-way ANOVA with Dunnett’s post-hoc test).

**Figure S10**: Mean fold-change levels compared to Sham of TNFα measured in the basal cell culture medium sampled prior to next exposure (day 28, prior to harvesting) of tissues to 16, 32 or 48 puffs of p-HTP Regular, p-HTP Intense aerosols or 1R6F smoke. Error bars represent standard deviation; n=3. Statistically significant changes from Sham for each timepoint are denoted by asterisks (*): *p≤0.05; **p≤0.005; ***p≤0.001; ****p≤0.0001 (two-way ANOVA with Dunnett’s post-hoc test).
